# A Novel Measure Inspired by Lyapunov Exponents for the Characterization of Dynamics in State-Transition Networks

**DOI:** 10.3390/e23010103

**Published:** 2021-01-12

**Authors:** Bulcsú Sándor, Bence Schneider, Zsolt I. Lázár, Mária Ercsey-Ravasz

**Affiliations:** 1 Department of Physics, Babes-Bolyai University, 400084 Cluj-Napoca, Romania; schbenc@gmail.com (B.S.); zsolt.lazar@ubbcluj.ro (Z.I.L.); 2 Network Science Lab, Transylvanian Institute of Neuroscience, 400157 Cluj-Napoca, Romania

**Keywords:** Lyapunov exponents, state-transition networks, time series analysis, dynamical systems

## Abstract

The combination of network sciences, nonlinear dynamics and time series analysis provides novel insights and analogies between the different approaches to complex systems. By combining the considerations behind the Lyapunov exponent of dynamical systems and the average entropy of transition probabilities for Markov chains, we introduce a network measure for characterizing the dynamics on state-transition networks with special focus on differentiating between chaotic and cyclic modes. One important property of this Lyapunov measure consists of its non-monotonous dependence on the cylicity of the dynamics. Motivated by providing proper use cases for studying the new measure, we also lay out a method for mapping time series to state transition networks by phase space coarse graining. Using both discrete time and continuous time dynamical systems the Lyapunov measure extracted from the corresponding state-transition networks exhibits similar behavior to that of the Lyapunov exponent. In addition, it demonstrates a strong sensitivity to boundary crisis suggesting applicability in predicting the collapse of chaos.

## 1. Introduction

Complex network theory has had many interdisciplinary applications in different domains of social sciences, epidemiology, economy, neuroscience, biology etc. [1]. In recent years different network approaches have been also developed for nonlinear time series analysis. For a detailed review see [2]. Proper mapping between a discrete time series and a complex network in order to apply the tools of network theory in an efficient manner is not a trivial question. In case of continuous-time dynamical systems it can be even more complicated. There are several approaches to this problem, here we mention three large categories [2]: (1) Proximity networks are created based on the statistical or metric proximity of two time series segments. The most studied variant of proximity networks are recurrence networks [3]. These have found many applications, in the characterization of discrete [4] and continuous dynamical systems [5,6], in the classification of medical signals [7], and in the analysis of two-phase flows [8]. A special version are joint recurrence networks, which were developed for the detection of synchronization phenomena in coupled systems [9]. (2) Visibility graphs capture the convexity of subsequent observations [10]. The methods of natural and horizontal visibility graphs belong to this class. Visibility graphs were used for the analysis of geophysical time series [11], for the characterization of seismic activity [12], two-phase fluid flows [13], and for algorithmic detection of autism [14]. (3) State-transition networks (STN) represent the transition probabilities between discretized states of the dynamics. These can be threshold-based networks [15] or ordinal partition networks [16]. These also have found many applications in the domain of biological regulatory networks [17], study of signals of chaotic circuits [18], electrocardiography [19], economic models [20] and climate time series [21]. STNs are in fact an equivalent representation of discrete-time finite-state Markov chains with a time-homogeneous transition matrix [22]. The adjacency matrix of the STN is the transition matrix of the Markov chain, and it is a right stochastic matrix (a real square matrix, with each row summing up to one [23]), having many known properties [22], see Section 2.1 for details. One of the frequently used entropy-type measures for characterizing Markov chains is the Kolmogorov–Sinai entropy [24].

While these approaches have been extremely useful - as shown by the wide range of applications, people have always concentrated on applying graph theory tools to obtain a new understanding of the dynamics, employing several traditional network measures [1,2]. Here we would like to take an inverse step and generalize a prominent measure from nonlinear dynamics theory on STNs. The Lyapunov exponent is one of the most used quantities for analyzing dynamical systems [25]. However, its estimation for time series, when the dynamical equations are not available, is seldom trivial [26]. Here we introduce an analogous measure on STNs that can become an effective measure also in time series analysis. Inspired by the theory of dynamical systems we look at the trajectory length of a system evolving over discrete time according to the transition probabilities defining its STN. We show that by associating the appropriate length measure to the transitions the combined ensemble and time average of the length of a single step yields the Kolmogorov–Sinai entropy. The new quantity, we name it Lyapunov measure, is defined in analogy to the Lyapunov exponent by estimating the variance of trajectory lengths during a random walk over the network. We find that the Lyapunov measure is able to distinguish between periodic and chaotic time series, and detect furthermore crisis-type bifurcations by presenting pronounced peaks in the vicinity of these parameters.

After a short description of the STNs, we present the new Lyapunov measure and its properties. We test and compare its behaviour with the Kolmogorov–Sinai entropy on a theoretical network model with cyclic properties, the discrete-time Henon map [27], and the continuous-time Lorenz system [28].

## 2. Results

### 2.1. State-Transition Networks

Mapping a dynamical system into an STN requires us to assign the different states of the dynamics to certain nodes of the network [15,16]. Directed edges of the network correspond to transitions between the discrete (or discretized) states of the system, characterized by the transition probability $p_{ij}$ from state *i* to *j*, where the probability of leaving the node equals to one (we have a right stochastic matrix),

(1) $$\sum_{j}p_{ij}=1\;,\;\;p_{ijk}=p_{ij}p_{jk}\;.$$

Trajectories consisting of several consecutive timesteps of the dynamics determine a path between distant nodes *i* and *k* of the STN, with the probability of selecting a particular path, e.g., i→j→k, given that the trajectory starts from node *i* is hence being given by the product of the respective transition probabilities. The transition probabilities, $p_{ij}$ introduced in Equation (1) are conditional by their definition assuming that the starting node of the transition is *i*. Therefore, the non-conditional probability of visiting edge i→j can be expressed by the Bayes-formula,

(2) $$q_{ij}=x_{i}p_{ij}\;,$$

where $x_{i}$ is the probability of residing in node *i*.

Analogously to geometric distance, one may assign a length $l_{ij}$ as weights to the respective edges [29], which takes low values for high probabilities and vice-versa:

(3) $$l_{ij}=-\ln p_{ij}\;,\;\;L_{ijk}=l_{ij}+l_{jk}\;.$$

The total length $L_{ijk}$ of such a i→j→k path is then given by the sum of lengths of the links along the path. A time series can hence be encoded by an STN as described above. Nodes of the network represent the spatial structure, while the time-like behavior is encoded by weighted and directed edges.

Mathematically, the weighted adjacency matrix of transition probabilities, ${\left(P\right)}_{ij}=p_{ij}$, determines the time evolution of an ensemble of trajectories on the STN:

(4) $$x\left(t+1\right)=P^{⊤}x\left(t\right)\;,\;\;\left|\left|x\left(t\right)\right|\right|=1\;,$$

where $x\left(t\right)={\left(x_{1}\left(t\right),\ldots ,x_{i}\left(t\right),\ldots ,x_{N}\left(t\right)\right)}^{⊤}$, and $x_{i}\left(t\right)$ denotes the probability of finding the system in state (node) *i* at time *t*. This process may also be seen as a time-homogeneous discrete-time Markov chain with a finite state space [24]. For STNs the stationary distribution is given by the renormalized eigenvector corresponding to the unit eigenvalue,

(5) $$P^{⊤}x^{*}=1\cdot x^{*}\;,\;\;x^{*}\left(t+1\right)=x^{*}\left(t\right)=x^{*}\;,\;\;\left|\left|x^{*}\right|\right|=1\;.$$

Note that since the evolution operator $P^{⊤}$ of the STNs considered here is a stochastic irreducible matrix [23], its largest eigenvalue is always 1 and the existence of the corresponding positive eigenvector is guaranteed by the Perron-Frobenius theorem [30]. For aperiodic transition matrices the long-term distribution is independent of the initial conditions, the system evolving over time to the stationary state $x^{*}$. In case of periodic solutions however the long-time averaged distribution is also given by the stationary solution,

(6) $$\lim_{t\to \infty }\frac{1}{t}\sum_{k}^{t}x\left(k\right)=x^{*}\;.$$

### 2.2. Lyapunov Measure

**Figure 1:**
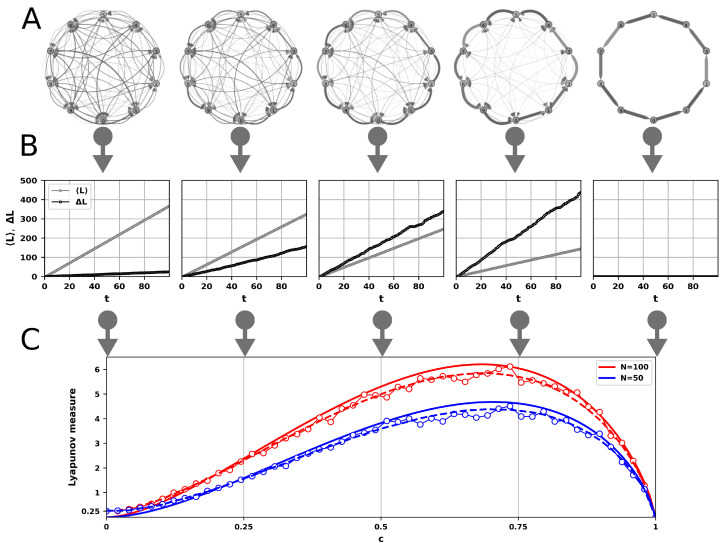
Dependence of the Lyapunov measure defined in Equation (14) on the cyclicity parameter, *c*, for complete graphs of different sizes, *N*, edges associated with probabilities defined in Equation (15) and lengths expressed by Equation (3). (**A**) Examples of graphs with N=10 nodes using different cyclicity parameters, *c*. The probabilities, $p_{ij}$, associated with edges are represented by their width. (**B**) Dependence of the mean, 〈L〉, and variance, ΔL, of trajectory length (see Equation (8)) on the number of steps, *t*, during simulated random walk on graphs similar to those in panel (**A**). The size of the networks is N=50. Data points correspond to ensemble averages of 1000 independent trajectories. (**C**) The Lyapunov measure as a function of the cyclicity parameter, *c*, for graphs similar to those in panel (**A**). Noisy lines with markers represent simulation results of random walk on these graphs. The measure is calculated according to Equation (14) using ensemble averages of 1000 trajectories. Each trajectory includes 1000 steps. Starting nodes are chosen randomly with uniform probability and the first 500 “thermalization” steps are ignored when estimating trajectory lengths. Dashed lines represent simulation results on similar graphs by estimating the variance of the edge length according to Equation (13) with 〈C〉=0. Continuous lines represent the analytical model (see Equation (20)).

In the context of dynamical systems theory, Lyapunov exponents are the best known quantities used to characterize the system’s behavior [25,26]. Here, we introduce an analogous quantity for STNs. Given a STN, one may define trajectories similarly to random walks on graphs: for any given initial state *i* the next state *j* is chosen randomly, using the transition probabilities $p_{ij}$. As described above, the weighted length of such a trajectory segment is given by Equation (3). By concatenating *t* subsequent segments one may construct an ensemble of different trajectories each of length

(7) $$L\left(t\right)=\sum_{k=1}^{t}l_{k}\;,$$

where $l_{k}$ is the length associated to the *k*th segment in the trajectory. For a large enough ensemble, in the limit of long times, both the average of the total path length 〈L〉 and its variance ΔL scales linearly with the total number of steps, *t* (see the Erdős-Rényi-type weighted random graphs in Figure 1A and the respective average lengths 〈L〉 and variance ΔL as a function of time in Figure 1B):

(8) $$\langle L\left(t\right)\rangle \propto t\;,\;\;\Delta L\left(t\right)=\langle L{\left(t\right)}^{2}\rangle -{\langle L\left(t\right)\rangle }^{2}\propto t\;.$$

As we shall see in the following the first observation allows for the measurement of the Kolomogorov–Sinai entropy [24] for STNs, while the second one enables us to define a novel network measure, similar in spirit to the Lyapunov exponents.

While the above scaling behavior offers an intuitive picture of the properties of average path length and its variance, an alternative approach allows a straightforward mathematical treatment and facilitates computation to a great extent. In the limit of infinitely large ensemble of infinitely long walks the problem reduces to a basic Markov process. Using the definition of the total trajectory length (7), one can easily show that the asymptotic behavior, that is when t→∞, of the ensemble averaged path length,

(9) $$\langle L\left(t\right)\rangle \to \langle l\rangle t\;,\;\;\lim_{t\to \infty }\langle l\left(t\right)\rangle =-\sum_{i,j}^{N}q_{ij}^{*}\ln p_{ij}=\langle l\rangle$$

grows linearly in time. The scaling factor, given by the average edge length of the STN, 〈l〉, weighted by the non-conditional occurrence probability, $q_{ij}^{*}=x_{i}^{*}p_{ij}$, of visiting the edge i→j in the asymptotic limit (compare Equation (2)), turns out to be equivalent with the Kolmogorov–Sinai entropy for Markov chains [24],

(10) $$S_{KS}=\lim_{t\to \infty }\frac{\langle L(t)\rangle }{t}=\langle l\rangle \;,$$

measuring the average entropy of transition probabilities, with respect to the stationary distribution $x_{i}^{*}$ given by Equation (5).

Similarly, due to the intrinsic diffusive nature of the envisioned process the variance of the trajectory length, ΔL, also grows linearly with the number of steps, *t*. The average squared path length,

(11) $$\langle L^{2}\left(t\right)\rangle \to \langle l^{2}\rangle t+{\langle l\rangle }^{2}t^{2}-{\langle l\rangle }^{2}t+\langle C\rangle t\;,\;\;\lim_{t\to \infty }\langle l^{2}\left(t\right)\rangle =\sum_{i,j}^{N}q_{ij}^{*}\ln^{2}p_{ij}=\langle l^{2}\rangle \;,$$

can be given in terms of the average lengths 〈l〉 and squared lengths $\langle l^{2}\rangle$, and of the asymptotic average of the correlation function $C(k,k^{\prime })$ for timesteps $k,k^{\prime }\to \infty$,

(12) $$\sum_{k\neq k^{\prime }}^{t}C\left(k,k^{\prime }\right)\to \langle C\rangle t\;,\;\;C\left(k,k^{\prime }\right)=\langle l\left(k\right)l\left(k^{\prime }\right)\rangle -\langle l\left(k\right)\rangle \langle l\left(k^{\prime }\right)\rangle \;.$$

The variance of the total path lengths

(13) $$\Delta L\left(t\right)=\langle L^{2}\left(t\right)\rangle -{\langle L\left(t\right)\rangle }^{2}=\sigma_{l}^{2}t+\langle C\rangle t\;,$$

is hence proportional to the variance of edge lengths for the whole network, $\sigma_{l}^{2}=\langle l^{2}\rangle -{\langle l\rangle }^{2}$, weighted according to transition frequencies during the stationary Markov process and the time averaged correlation function 〈C〉.

Traditional Lyapunov exponents measure the exponential divergence rate of initially close-by trajectories during the time evolution of a dynamical system. For STNs, we can define a network measure bearing similarities with the Lyapunov exponents by considering the scaling of the length difference of pairs of random paths started from the same initial node of the network. As a further simplification, we define the Lyapunov measure for STNs using a proportional quantity, the variance of the total length of single trajectories,

(14) $$\Lambda =\lim_{t\to \infty }\frac{\Delta L(t)}{t}=\sigma_{l}^{2}+\langle C\rangle \;.$$

For uncorrelated processes, viz when 〈C〉=0, we can estimate the mean 〈L〉 and the variance ΔL of the trajectory lengths by the overall mean 〈l〉 and variance $\sigma_{l}^{2}$ in the length “covered” in a single step, a methodology which will be first tested for random networks.

#### 2.2.1. Properties of the Lyapunov Measure

The STN analog of limit cycles and strange attractors in dynamical systems would be circular networks and high degree networks, respectively. Let us investigate the dependence of a STN’s Lyapunov measure, Λ, on the degree of cyclicity of the network. We shall build our description on a simple theoretical model consisting in a complete directed graph of *N* nodes identified by their label i∈0,1,…,N−1. In order to study the effect of cyclicity the transition probabilities are associated to the edges in three steps: first, to each of the N(N−1) links we assign a random $u_{ij}$ value distributed uniformly between zero and one; second, we rescale these probabilities such that each node has a privileged target and source node with transition probabilities:

(15) $$p_{ij}\left(c\right)=\left\{\begin{array}{cc} c+\left(1-c\right)u_{ij}\;,\; & \mathrm{if}\;j=(i+1)\;\mathrm{mod}\;n\;,\\ \left(1-c\right)u_{ij}\;,\; & \mathrm{if}\;j\neq (i+1)\;\mathrm{mod}\;n\;, \end{array}\right.$$

where “mod” denotes the modulo operation and c∈[0,1] is the parameter quantifying the cyclicity of the graph (see Figure 1A). At c=1 we have a purely cyclic graph where each node is only linked to the next node in the list while for c=0 we retain the original $u_{ij}$ weights. As a last step, we normalize the outgoing weights according to Equation (1). Computing the average and the variance for an ensemble of trajectory lengths, started from random initial nodes, we obviously recover the theoretically calculated linear scaling behavior (compare Equations (9) and (13)).

The results of the simulation implementing Equation (14) are represented by the noisy lines with markers in Figure 1C. There is an apparent dependence on the size of the network. However, a complete graph with all links equivalent (c=0) with weights distributed uniformly yields Λ=1/4 irrespective of the size of the network. A simple cycle with all nodes having a single incoming and outgoing link (c=1) eliminates all randomness and hence the variance based Lyapunov measure goes to zero. For intermediate values of the cyclicity parameter, *c*, the Lyapunov-measure evolves smoothly exhibiting a maximum somewhere in the upper half of the unit interval.

#### 2.2.2. Analytical Study of the Lyapunov Measure

For a conceptual grasp of the behavior shown in Figure 1C we further simplify our model. In a fully connected graph of size *N* all $l_{i}$-s in Equation (7) can be regarded as identical random variables, hence

(16) $$\Lambda =\sigma_{l}^{2}\;$$

where $\sigma_{l}^{2}\equiv \langle l^{2}\rangle -{\langle l\rangle }^{2}$ is the variance of the variables. We can work with a single vertex and extrapolate the results to all vertices. One neighbor of this “generic” vertex is privileged as it is the next in the cycle (see Figure 1A). Let us assume that the rest of the N−2 neighbors are equivalent (their links have equal weights):

(17) $$p_{i}=\left\{\begin{array}{cc} p\;, & \mathrm{if}\;i=1\;,\\ (1-p)/n\;, & \mathrm{if}\;i=\bar{2,N-1}\;, \end{array}\right.$$

where n=N−2, and with the cyclicity parameter c∈[0,1] the probability $p=\frac{1}{n+1}+\frac{n}{n+1}c$ will change between 1/(n+1) (all links equivalent) and one (fully cyclic). The length of link *i* is set to $-\ln p_{i}$, the probability of choosing that link is $p_{i}$:

(18) $$\langle l\rangle =-\sum_{i}p_{i}\ln p_{i}=-p\ln p-\left(1-p\right)\ln\frac{1-p}{n}\;,$$

(19) $$\langle l^{2}\rangle =\sum_{i}p_{i}\ln^{2}p_{i}=p\ln^{2}p+\left(1-p\right)\ln^{2}\frac{1-p}{n}\;,$$

(20) $$\Lambda =p\left(1-p\right)\ln^{2}\frac{np}{1-p}\;.$$

The 1/4 offset at c=0 is a consequence of the fixed transition probability values in Equation (17). Appendices Appendix A and Appendix C account for this offset. A detailed investigation of the maximum is under Appendix B.

### 2.3. Time Series Analysis with STNs

The random network model in the previous section has the benefit of offering a basic understanding of the Lyapunov measure. In order to test the scope of its applicability we apply it to the well-known Henon map and the Lorenz system and compute Λ for the STNs constructed for time series with different control parameters. We show that the network measure is able not only to distinguish between periodic and chaotic regimes but also presents pronounced peaks before crisis-type bifurcations. To that end we need to map “real-world” time series to STNs. In this section we introduce a methodology which is generic enough to be applied both on discrete- and continuous-time dynamical systems.

#### Construction of STNs

To construct the STN corresponding to the dynamics of the time series generated by a dynamical system one needs to discretize space and time adequately. In case of discrete-time systems time is inherently defined in terms of integer-valued timesteps. For continuous-time systems this step is however not as straightforward. A time sampling with a constant sampling rate may not be beneficial for all cases since the trajectory may evolve with various speeds in the phase space, having segments where it slows down while at other parts advancing abruptly orders of magnitude further within the same time interval. A too high sampling frequency would lead to a very high number of self-loops in the STN, since for any finite spatial mesh there exist several consecutive time points which fall within the same grid cell. On the other hand, a very low sampling rate leads to the loss of time correlations. Here we propose to use the well-defined method of Poincare sections, which naturally generates the equivalent map, i.e., the discrete-time version of the dynamical system. The Poincare map can be constructed by tracking the consecutive unidirectional intersection points of the trajectory and the Poincare surface, a 2D surface in case of 3D systems.

On the other hand, for an STN one needs to assign the nodes of the network to different states during the dynamics. One may hence discretize the phase space by dividing it into equal sized bins, corresponding to the nodes of the network. Here we consider the effective phase space of the system, viz the largest rectangle in the phase plane of the map/Poincare map which fully covers the attractor, and construct a mesh, each of the newly created bins defining a coarse-grained state of the original dynamical system. Directed edges of the network correspond then to transitions between the discrete states of the phase space, characterized by the transition probabilities $p_{ij}$.

**Figure 2:**
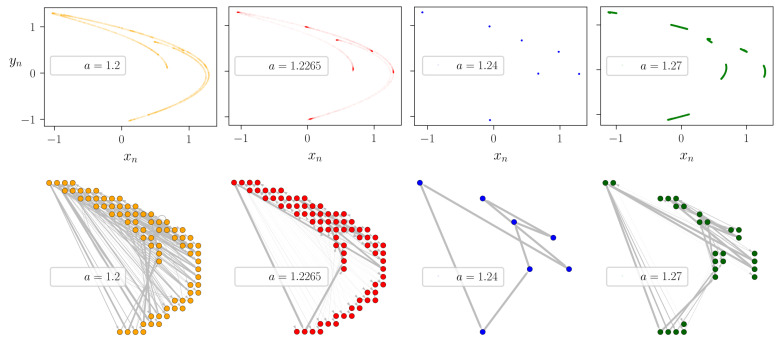
Constructing state-transition networks for the Henon-map. **Top row:** Chaotic and periodic attractors of the Henon-map in the $\left(x_{n},y_{n}\right)$ plane for a=1.220/1.2265/1.24/1.27 (orange/red/blue/green), respectively. **Bottom row:** The corresponding state-transition networks with a spanning layout respecting the $\left(x_{n},y_{n}\right)$ positions of the nodes in the phase plane of the system. For illustration, the widths of edges are set proportionally to the occurrence of the respective transitions during the dynamics. To construct the STN $n_{\max}=10^{4}$ steps long trajectories are started from randomly chosen initial conditions while discarding the first $n_{\mathrm{trans}}=10^{3}$ transient steps. For discretization of the effective phase plane a 20×20 mesh is constructed.

**Figure 3:**
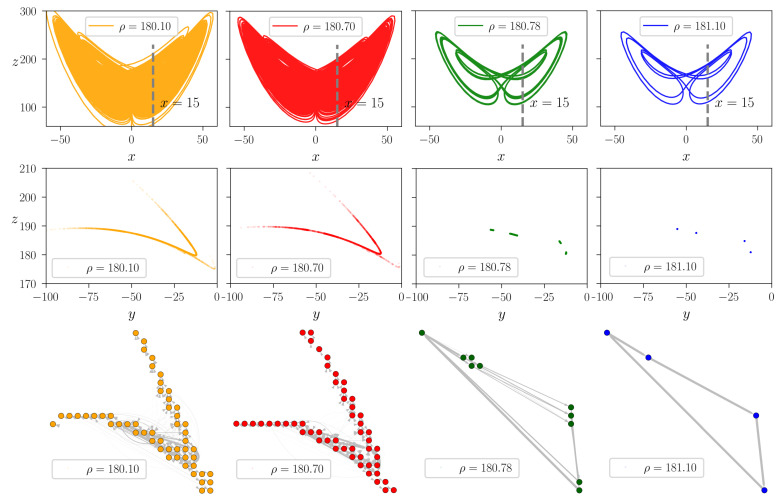
Constructing state-transition networks for the Lorenz system. **Top row:** Chaotic and periodic attractors of the Lorenz in the (z,y) plane for ρ=180.10/180.70/180.78/181.10 (orange/red/green/blue), respectively. **Middle row:** The x=15, $\dot{x}<0$ Poincare sections of the attractors (see the top panels). **Bottom row:** The corresponding state-transition networks with a spanning layout respecting the (z,y) positions of the nodes in the Poincare sections of the system. For illustration, the widths of edges are set proportionally to the occurrence of the respective transitions during the dynamics. For illustrating the Poincare maps $t_{\max}=5000$ time unit long trajectories are used, discarding $t_{\mathrm{trans}}=300$ long transients. For discretization of the effective phase plane a 20×20 mesh is constructed.

To illustrate the methodology we present examples of different attractors with the corresponding STNs for the discrete-time Henon map [27] and the continuous-time Lorenz system [28] (see Figure 2 and Figure 3). The two systems are briefly surveyed in Section 3.1. For the Henon-map we use $n_{\max}=10^{4}$ steps long trajectories started from randomly chosen initial conditions while discarding the first $n_{\mathrm{trans}}=10^{3}$ transient steps to let the dynamics settle onto the attractor of the system. As a function of the *a* parameter the attractor may have either a fractal structure of folded filaments or it may consist of discrete points separated in the phase plane (see the top panels of Figure 2). For the Lorenz system we used the method of Poincare sections, with the Poincare surface being a predefined 2D plane. As an example, in Figure 3 we illustrate the projection of the x=15 Poincare plane by a dashed line in the (z,x) phase plane (top row). The corresponding Poincare map is constructed using $t_{\max}=5000$ time unit long trajectories, discarding the initial $t_{\mathrm{trans}}=300$ time units. The choice of the Poincare section does not effect the results qualitatively. For comparison, a second, perpendicular Poincare section, defined by y=0, have also been tested (not shown here). We found that the results are robust with respect to the choice of the Poincare plane. The topology of the resulting Poincare sections also reflect faithfully the structure of the attractors (middle row).

For both the Henon map and Lorenz system we selected parameters corresponding to qualitatively different dynamical regimes: traditional chaotic attractors with extended fractal filaments (orange and red colors), partially predictable chaotic attractors with small patches of Cantor-sets [31] (green color), and individual points for periodic attractors (blue).

The STNs are constructed using a 20×20 mesh in the phase planes of the respective maps. For computing the transition probabilities $p_{ij}$ we generate long discrete-time trajectories as described above and count the number of i→j transitions along these trajectories. The $p_{ij}$ probabilities are finally given by normalizing the number of jumps between consecutive states according to Equation (1). Chaotic dynamics generates densely wired graphs (left columns of Figure 2 and Figure 3) and widely distributed global transition frequencies $\propto q_{ij}^{*}$ (as illustrated by the width of the network connections), in contrast to periodic motion for which the network collapses to a simple cyclic chain of edges and vertices (right column of Figure 2 and Figure 3). Though, seemingly two of the selected chaotic attractors are identical (see the orange and red attractors in Figure 2 and Figure 3), looking more closely one realises that for the red colored STNs the global transition probabilities (the widths of the edges) are more heterogeneous than for the orange colored STNs, allowing for a more probable cyclic path in the network. Note that the random network model introduced in Section 2.2.1 resembles this network structure for large (but smaller than 1) cyclicity parameters, e.g., when c∼0.75.

### 2.4. Network Measures

**Figure 4:**
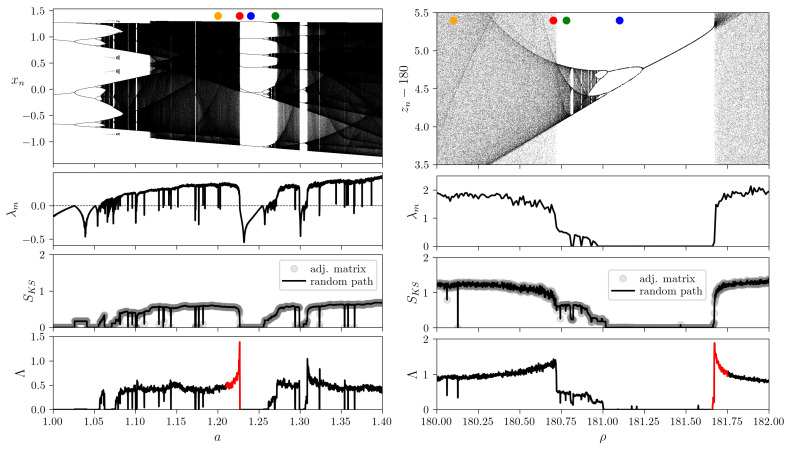
Network measures for STNs generated from time series of discrete- and continuous-time dynamical systems. (**Left**) Results for the Henon-map as function of the a∈[1,1.4] control parameter. (**Right**) Results for the Lorenz system, for the ρ∈[180,182] parameter interval. *From top to bottom*: Bifurcation diagrams, largest Lyapunov exponents $\lambda_{m}$, Kolmogorov–Sinai entropy $S_{KS}=\langle l\rangle$, and Lyapunov-type network measure Λ=ΔL/t for the same parameter intervals as for the bifurcation diagrams. The colored dots denote the parameter values for which the attractors, Poincare maps and STNs are shown, respectively, in Figure 2 and Figure 3. To construct the STNs the Henon-map is iterated for 3 × 10${}^{4}$ timesteps omitting the initial 1000 transient steps, respectively for the Lorenz system 5000 timeunits were used ignoring the first 500 units long transients. The Kolmogorov–Sinai entropy is computed both using the adjacency matrix of global transition probabilities $q_{ij}^{*}$ (compare Equations (9) and (10)) and by generating and ensemble of random paths using the same number of steps as for the Lyapunov measure. The Lyapunov network measure, defined by Equation (14), is calculated over an ensemble of 100 random trajectories of $10^{4}$ steps, neglecting the initial 5000 steps (see Section 3.3). As an example of boundary-crisis precursor, a single peak in the Lyapunov measure is colored in red for both systems.

To characterize the network with a single scalar parameter we compare the two STN measures discussed in Section 2.2. First, we adopt here the well-known Kolmogorov–Sinai (KS) entropy for Markov chains, as defined by Equation (9). Second, we compare the above introduced novel measure (14) similar in idea to the well-known Lyapunov exponents. As a basis of comparison we also provide the bifurcation diagrams of the Henon and Lorenz systems, together with the classical largest Lyapunov exponent $\lambda_{m}$ (see Figure 4).

As changing the control parameters *a* and ρ, for the Henon map (21) and for the Lorenz system (22) respectively, both exhibit a whole series of complex bifurcations (see the top panels of Figure 4). These bifurcation scenarios are, as expected, faithfully reflected by the largest Lyapunov exponent values: $\lambda_{m}<0$ and $\lambda_{m}=0$ for periodic behavior of the Henon map and Lorenz equations, respectively, while $\lambda_{m}>0$ corresponding to chaotic dynamics in both cases.

The Kolmogorov–Sinai entropy, $S_{KS}$, is defined for the STNs obtained from the dynamical systems for the actual control parameter values. Here we implement both the original definition based on the global transition probability matrix $q_{ij}^{*}$ and the algorithm based on generating random trajectories in the STN and measuring their linear scaling factor with time (compare Equations (9) and (10)). As expected the exact matching of the two definitions is reflected by the numerical results as well (see Figure 4): $S_{KS}=0$ denoting exactly cyclic networks, low positive values of $S_{KS}$ correspond to partially predictable chaotic motion, while chaotic dynamics generates high entropy networks.

The Lyapunov measure, Λ, is computed here based on the estimation of the variance in trajectory lengths for large ensembles of random paths, according to Equation (14) (see Section 3.3 for details on ensemble statistics). In the examined parameter region, while bearing many similarities with Kolmogorov–Sinai entropy, the Lyapunov measure exhibits interesting behavior in the vicinity of crisis-type bifurcations points (abrupt disappearance or reduction in size of the attractor, viz in the size of the black shaded regions in the bifurcation diagram) [32]. Approaching the crisis from the direction of the chaotic region Λ presents an abrupt peak, forecasting in some sense the collapse of the chaotic attractor (see the red colored sharp peaks in the bottom panels of Figure 4).

As it is demonstrated using the random network model in Section 2.2.1, the Lyapunov measure has a maximum point with respect to the cyclicity parameter (see also Appendix B). The appearance of the peaks is closely related to this special cyclic topology in real systems as well (as shown in Appendix D). The height of the peaks is furthermore boosted by the correlations of edge lengths along the paths of random walks (see Equation (14)).

Interestingly these precursor peaks seem to be more pronounced for boundary crisis than for the case of interior crisis (compare for example the red-colored peak of the Lorenz system with the peak around the parameter value denoted by the red dot).

Our results, summarized in Figure 4, demonstrate that STNs can encode all the relevant information about the dynamics of the system. For chaotic dynamics with positive maximal Lyapunov exponent $\lambda_{m}>0$ the network measure Λ is also positive. For periodic motion both quantities are zero, $\lambda_{m}=\Lambda =0$. Interestingly, while the Lyapunov exponent decreases when approaching the bifurcation point where chaos disappears, the here introduced Lyapunov measure shows a diverging tendency.

## 3. Materials and Methods

### 3.1. Discrete- and Continuous-Time Dynamical Systems

The Henon-map is considered as a prototype system for studying bifurcations and chaos in discrete-time dynamical systems [32]. The mapping from state $\left(x_{n},y_{n}\right)$ to $\left(x_{n+1},y_{n+1}\right)$ is defined by

(21) $$x_{n+1}=1-ax_{n}^{2}+y_{n}\;,\;\;y_{n+1}=bx_{n}\;.$$

The effect of the nonlinear term $x_{n}^{2}$ in the dynamics may be tuned by changing parameter *a*, hence in most of the studies it is considered the control parameter, while choosing b=0.3 standard value for the other parameter. As a function of the *a* parameter the attractor may have either a fractal structure of folded filaments or it may consist of discrete points separated in the phase plane (see the top panels of Figure 2).

The Lorenz system is probably the most known continuous-time dynamical systems exhibiting chaos on a butterfly-shaped attractor. Being a 3D system defined as

(22) $$\dot{x}=\sigma \left(y-x\right)\;,\;\dot{y}=x\left(\rho -z\right)-y\;,\;\dot{z}=xy-\beta z\;,$$

it is one of the simplest examples which allows for the presence of chaotic behavior. While we consider the standard σ=10 and β=8/3 parameters, we select with ρ∈[180,182] a parameter interval for which one finds not only chaotic and periodic behaviors, but also partially predictable chaos (PPC), as introduced in [31]. The respective attractors are illustrated in Figure 3.

### 3.2. Phase-Space Discretization and Poincare Sections

The binning of the phase space is a cornerstone in the construction of STN networks. For a low number of bins one loses information regarding the dynamical states of the system. On the other hand for a too high resolution the STN collapses to chain-like networks even for an otherwise complex dynamics, requiring exponentially long time series to overcome the statistical unreliability of the data. For chaotic time series, the number of nodes of the obtained STN increases with the binning resolution. Yet the measure proposed here turns out to be reliable since it is a monotonous function of the number of nodes (compare Equation (20) and Figure 1). The choice of the Poincare section also does not affect the results qualitatively. For comparison, two perpendicular Poincare sections have been tested, defined by x=15 and y=0, respectively. We found that the results are robust with respect to the choice of the Poincare plane used for the construction of the STN.

### 3.3. Ensemble Averages and Asymptotic Behavior

**Figure 5:**
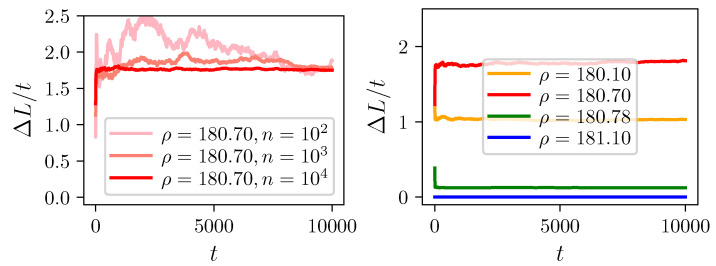
Lyapunov measure: variance of path lengths for random trajectories using the STNs generated for the Lorenz system. (**Left**) Averaged over an ensemble of *n* trajectories (see the legend) for the chaotic regime with ρ=180.7. (**Right**) Averaged over $n=10^{4}$ trajectories for chaotic and periodic dynamics using ρ=180.10/180.70/180.78/181.10, respectively (orange/red/green/blue).

Given a STN, the Lyapunov measure (14) is computed numerically by constructing random trajectories of total length $L_{i}\left(t\right)$ at time *t*, and measuring their variance over an ensemble of *n* paths, i=1,…,n. For a large enough ensemble the ΔL/t converges to a steady state value, termed here as the Lyapunov network measure and denoted by Λ. For the STNs obtained from the Lorenz-system generated time series for ρ=180.7 the fluctuations become relatively small for an ensemble average computed over $n>10^{3}$ random trajectories and for t>5000 time units (see the left panel of Figure 5). In order to reduce the fluctuations one may further average over the stationary ΔL/t values in time. Different dynamical behaviors correspond to separated plateaus in the time series of ΔL/t, leading to well-defined and different Lyapunov-measure values for four parameter settings presented in Figure 3 (compare the right panel of Figure 5).

## 4. Discussion

Complex dynamics in continuous phase space and time bears similarities and differences compared to those occurring on networks. Inspired by the utility of the Lyapunov exponent in dynamical systems theory we introduced a somewhat analogous network measure for STNs based on the Kolmogorov–Sinai entropy for graphs. The random STN model shows that using the variance instead of the mean length, equivalent with the Kolmogorov–Sinai entropy, contributes to a surge in the measure as we approach cyclicity (see Figure 1C). Our analytical study explains the important properties of the new measure as they manifest in the case of random networks. In order to assess the connection between the new measure and the classical Lyapunov exponent we also introduced a novel procedure for converting time series to STNs. Our examples include both discrete-time (Henon-map) and continuous-time (Lorenz) systems. The Kolmogorov–Sinai entropy of the so obtained Henon/Lorenz STNs reproduces the control-parameter dependence of the Lyapunov exponent of the corresponding dynamical systems. The newly introduced Lyapunov measure, however, exhibits an additional and unexpected property. It appears to be sensitive to boundary crisis where a chaotic attractor is suddenly created or destroyed (see bottom panels of Figure 4). This extreme jump in the measure during crisis is due to the cumulation of the basic increase experienced also with random networks (see Figure 1C) and the effect of correlations due to the heterogeneity of the Henon/Lorenz STNs. In present paper the Lyapunov measure for these networks was estimated based on trajectory simulations. However, accounting for the correlations as introduced in Equation (12) should be possible based solely on the transition probabilities, $p_{ij}$. In this sense, similarly to the case of the Lyapunov exponent, it may allow two slightly different but –for the case of ergodic systems– equivalent interpretations: one may either follow trajectories and compute the quantities of interest along them or determine the natural/stationary distributions underlying the dynamics and calculate spatial averages according to these probabilities [32]. The pronounced peaks during boundary crisis suggests possible applications for forecasting transitions from chaos to periodicity.

The proposed method can be applied to any stationary time series and any dynamical system with attractors. It might not always provide as relevant output as for the two examples given in the paper. We expect, however, similar results for most of the commonly studied systems. In case of multistable dynamical systems the Lyapunov measure has to be computed separately for each attractor, similarly to the traditional Lyapunov exponent. For non-stationary time series the proposed methods and quantities may need further developments.

## Figures and Tables

**Figure A1:**
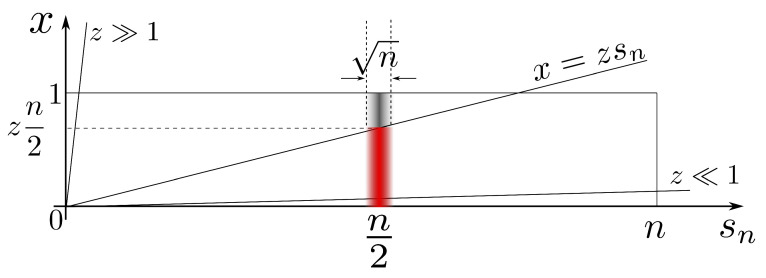
Depiction of the essentials behind Equation (A5).

**Figure A2:**
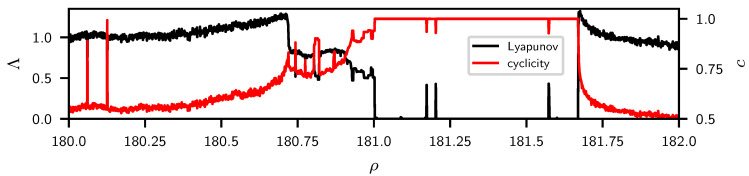
Semianalytic estimation of the cyclicity, *c*, and Lyapunov measure, Λ, of the Lorenz STNs created according to the procedure described in Section 2.3. The values of the mean edge length, 〈l〉, are computed following the definition of the Kolmogorov–Sinai entropy for STNs expressed by Equation (9). The cyclicity parameter is estimated by inverting numerically the 〈l〉(c) relationship in Equation (18). As a last step, the Lyapunov measure was approximated using the analytical expression from Equation (20). The random walk simulation counterpart of the image is presented in the bottom right panel of Figure 4. For more details, refer to Appendix D.

## Data Availability

The Python code developed to construct the state-transition networks and compute Lyapunov measure are available online at https://github.com/schbence/stn-lyapunov.

## Appendix A. Offset at *c* = 0 in the Lyapunov Measure According to the Random Network Model

The 1/4 offset at c=0 is a consequence of the fixed transition probability values in Equation (17) as compared to the uniformly distributed random values prescribed by Equation (15). The offset can be recovered by replacing the set 1/(N−1) values by random numbers distributed uniformly between 0 and 2/(N−1). This distribution is asymptotically, N→∞, correct for sum-normalized random numbers (see Appendix C). The searched variance can be approximated by

(A1) $$\sigma_{l}^{2}=\frac{2}{\alpha }\bar{p\ln^{2}p}-\frac{4}{\alpha^{2}}{\bar{p\ln p}}^{2}\;,$$

where p=αx, α=2/(N−1) and p.d.f(x) = 1. Making use of the identities

(A2) $$\int_{0}^{1}x\ln x\;dx=\int_{0}^{1}x\ln^{2}x\;dx=\frac{1}{4}$$

we get Λ=1/4.

## Appendix B. Location and Size of the Maximum in the Lyapunov Measure According to the Random Network Model

The maximum can be studied via the stationarity criterion dΛ/dp=0 leading to the equation

(A3) $$\ln\left(\frac{p}{1-p}\right)+\frac{2}{1-2p}=-\ln n\;.$$

Both terms on the lhs can match the divergence on the rhs either by p↘0 or p↘1/2, respectively. In the former case the required asymptotic dependence becomes $p∼1/e^{2}n$ which is below the 1/(n+1) lower limit prescribed by Equation (17), therefore not acceptable. However, the divergence of the second term can be achieved by c≈p∼1/2+1/lnn yielding a maximal Lyapunov-measure diverging as

(A4) $$\Lambda_{\max}=\frac{1}{2}\ln^{2}n+O\left(\ln n\right)\;.$$

## Appendix C. Distribution of Uniform Random Numbers Normalized over Large Sets

Let $x_{0},x_{1},\ldots ,x_{n}\in \left[0,1\right)$ be uniformly distributed random variables. The distribution of the normalized variables $x_{j}/\sum_{i=0}^{n}x_{i}$ is the same for all j=0,1,…,n. For very large *n* the correlation between the numerator and the denominator tends to disappear therefore we can as well look at the distribution of $z=x/s_{n}$ where $s_{n}=\sum_{i=1}^{n}x_{i}$ and $x=x_{0}$. According to the central limit theorem the distribution of $s_{n}\in \left[0,n\right)$ can be approximated by a Gaussian centered at n/2 and a width growing only as $\sqrt{n}$ (see Figure A1). The cumulative distribution function of *z* in the limit of large *n* becomes

(A5) $$c.d.f\left(z\right)=\int_{x<zs_{n}}dx\;ds_{n}\;p.d.f\left(x\right)p.d.f\left(s_{n}\right)=\left\{\begin{array}{cc} z\frac{n}{2}\;,\; & z\leq \frac{2}{n}\\ 1\;,\; & z>\frac{2}{n} \end{array}\right.\;,$$

yielding

(A6) $$p.d.f\left(z\right)=\frac{d}{dz}c.d.f\left(z\right)=\left\{\begin{array}{cc} \frac{n}{2}\;,\; & z\leq \frac{2}{n}\\ 0\;,\; & z>\frac{2}{n} \end{array}\right.\;.$$

## Appendix D. Insight into the Emergence of Precursor Peaks

The surge in the Lyapunov measure as we approach the collapse of chaos (see the region in red in the bottom panels of Figure 4 is a cumulative result of three factors: (i) the random network model in Section 2.2.1 shows that as cyclicity grows the measure attains a maximum (see Figure 1C); (ii) below we provide evidence for a superlinear growth in the cyclicity itself close to the bifurcation point; (iii) nontrivial correlations (see Equation (12)) present in real STNs can further boost the effect. Within the presented conceptual framework cyclicity can be quantitatively defined only through the random network model of Section 2.2.1. Its scope can be extended to general networks through Equations (9) and (18). Following Equation (9) we can compute the Kolmogorov–Sinai entropy, ergo, the mean length 〈l〉 of an edge for any STN. By inverting the relationship 〈l〉(c) from Equation (18) we can extract information on the cyclicity, *c*, of these networks. Subsequently, the Lyapunov measure can be estimated from Equation (20). There is an inherent mismatch between the two approaches to the mean lengths formalized in Equations (10) and (18). As opposed to a general STN the analytical model assumes a complete graph making the degree of the nodes and the size of the network coincide. This discrepancy can be to some extent bridged by using the average node degree as *n* when inverting the relationship in Equation (18). The results depicted in Figure A2 are the counterparts of those shown in the bottom right panel of Figure 4.
